# A Sub-Microscopic Gametocyte Reservoir Can Sustain Malaria Transmission

**DOI:** 10.1371/journal.pone.0020805

**Published:** 2011-06-14

**Authors:** Stephan Karl, David Gurarie, Peter A. Zimmerman, Charles H. King, Tim G. St. Pierre, Timothy M. E. Davis

**Affiliations:** 1 School of Physics, The University of Western Australia, Crawley, Western Australia, Australia; 2 Department of Mathematics, Case Western Reserve University, Cleveland, Ohio, United States of America; 3 The Center for Global Health and Diseases, Case Western Reserve University, Cleveland, Ohio, United States of America; 4 School of Medicine and Pharmacology, The University of Western Australia, Fremantle Hospital, Western Australia, Australia; Kenya Medical Research Institute - Wellcome Trust Research Programme, Kenya

## Abstract

**Background:**

Novel diagnostic tools, including PCR and high field gradient magnetic fractionation (HFGMF), have improved detection of asexual *Plasmodium falciparum* parasites and especially infectious gametocytes in human blood. These techniques indicate a significant number of people carry gametocyte densities that fall below the conventional threshold of detection achieved by standard light microscopy (LM).

**Methodology/Principal Findings:**

To determine how low-level gametocytemia may affect transmission in present large-scale efforts for *P. falciparum* control in endemic areas, we developed a refinement of the classical Ross-Macdonald model of malaria transmission by introducing multiple infective compartments to model the potential impact of highly prevalent, low gametocytaemic reservoirs in the population. Models were calibrated using field-based data and several numerical experiments were conducted to assess the effect of high and low gametocytemia on *P. falciparum* transmission and control. Special consideration was given to the impact of long-lasting insecticide-treated bed nets (LLIN), presently considered the most efficient way to prevent transmission, and particularly LLIN coverage similar to goals targeted by the Roll Back Malaria and Global Fund malaria control campaigns.

Our analyses indicate that models which include only moderate-to-high gametocytemia (detectable by LM) predict finite eradication times after LLIN introduction. Models that include a low gametocytemia reservoir (requiring PCR or HFGMF detection) predict much more stable, persistent transmission. Our modeled outcomes result in significantly different estimates for the level and duration of control needed to achieve malaria elimination if submicroscopic gametocytes are included.

**Conclusions/Significance:**

It will be very important to complement current methods of surveillance with enhanced diagnostic techniques to detect asexual parasites and gametocytes to more accurately plan, monitor and guide malaria control programs aimed at eliminating malaria.

## Introduction

Recent regional roll back of malaria from Zanzibar, Eritrea, Ethiopia and Rwanda has been achieved by up-scaled and improved malaria control efforts through distribution of long lasting insecticide treated bed nets (LLIN) and effective treatment with artemisinin combination therapy (ACT) [1]–[3]. These successes plant new hope for malaria elimination given necessary levels of support [4]. In the progress report of the UN Secretary-General's Special Envoy for Malaria from 2009, it was announced that the continent of Africa now had surpassed average LLIN coverage of 40% with numerous African nations exceeding 60% [5]. In the malaria-endemic Southwest Pacific region, LLIN coverage rates of 70% have been reported from Papua New Guinea (PNG) and the Solomon Islands [6]. The importance of continued surveillance of progress towards the goal of near zero preventable deaths from malaria by 2015 has been stressed [5]. However for successful economic and strategic planning of large-scale malaria control, it is essential to be able to predict how long an intensified malaria control effort will have to be sustained before the threat of resurgent malaria transmission is removed.

Mathematical approaches, mainly based on fundamental work by Ross [7] and Macdonald [8], have been used to model malaria transmission and to predict the impact of malaria control during the malaria elimination campaigns in the 20th century [9]. These models utilize a population-based methodology whereby the relevant human population is divided into several subpopulations representing different states of infection. The subpopulations are usually called Susceptible, Exposed/Latent, Infective and Immune and they are connected by appropriate transitions [10]–[16]. Purely susceptible and immune states are rarely attained and therefore can be combined into a single ‘partially immune - partially susceptible’ subpopulation. The most convenient way to estimate the sizes of the subpopulations is to rely on observations of asexual parasite and gametocyte forms in peripheral blood. Since gametocytes are the sole parasite form possessing the ability to transmit malaria through the mosquito vector, only hosts harbouring these forms are part of an infective subpopulation. However, the fraction of individuals with detectable asexual parasites and gametocytes is dependent on the method of diagnosis. It has been estimated that approximately 50% of all infections with asexual parasites are not detected by standard light microscopic (LM) examination of thick blood films while for gametocytes, the percentage of false negative observations can be as high as 90% [17]. In a Kenyan study, for example, Bousema et al. reported that gametocyte prevalence observed by LM and quantitative nucleic acid sequence based amplification (QT-NASBA) were 22.3% and 91.1%, respectively [18], while a gametocyte prevalence of 7.6% by LM contrasted with 47% by magnetic deposition microscopy in a PNG survey [19]. Furthermore, recent studies have provided evidence that individuals who are not gametocyte positive by LM can still be infective. Gametocyte densities detectable by LM are usually reported to be >5–10 µL^−1^ but molecular and magnetic fractionation methods detect gametocytes at densities as low as 0.1 µL^−1^ [18], [20].

The differences between gametocyte rates detected using advanced methods and LM as well as improved data on the relationship between submicroscopic gametocyte densities and mosquito infection may have substantial consequences for our understanding of malaria epidemiology. Whereas low-level gametocytemia corresponds to reduced likelihood of mosquito infection, the contribution that microscopically non-identifiable gametocyte carriers make to malaria transmission is still considerable [21]–[23]. Mathematical models which use gametocyte rates determined by LM can considerably underestimate the size of the infective compartment and thus the required scale and duration of malaria control needed to achieve elimination. There is a risk of premature termination of control measures followed by resurgence of disease.

To examine the effect of a subpopulation harboring submicroscopic gametocytes on predicted malaria transmission and predicted impact of malaria control two mathematical models were compared and contrasted. The first model (M1) does not include any observations of submicroscopic asexual parasites or gametocytes. The second model (M2) includes submicroscopic data. The approach presented here is substantially different from the classical Ross-Macdonald setup as it attempts to account for superinfection and for the differences in susceptibility and infectivity of the subpopulations. To estimate model parameters, available data from a low and a high transmission environment [24]–[27], as well as individual cases involved in malaria therapy (MT) studies [19], [22], [23], [28]–[37], were used. Several hypothetical control experiments simulating malaria control based on the distribution of LLIN were conducted using the calibrated models.

The present study shows that mathematical models based on field data collected by LM alone are likely to deliver overly optimistic predictions. Planning malaria control and monitoring the progress of interventions aimed at eliminating malaria would therefore benefit significantly from wide application of improved diagnosis of asexual parasites and especially gametocytes.

## Methods

### Model description

All symbols used in the models presented in this study are explained in Table 1.

**Table 1:** Definition of symbols which were used in the present study.

Symbol/Unit	Definition
*σ* [d^−1^]	mosquito survival through extrinsic incubation period
*μ* [d^−1^]	mosquito mortality
*ω* [d^−1^]	bites per mosquito in 24 h
*τ* [d]	mosquito latency period
*γ* [d^−1^]	rate for gametocyte maturation
*r* [d^−1^]	recovery rate from microscopically detectable gametocytemia (including superinfection)
*ρ* [d^−1^]	recovery rate from submicroscopic gametocytemia (including superinfection)
*r_0_*[d^−1^]	natural recovery rate from microscopically detectable gametocytemia (without superinfection)
*ρ_0_*[d^−1^]	natural recovery rate from submicroscopic gametocytemia (without superinfection)
*h* [d^−1^]	human biting rate
EIR[a^−1^]	entomological inoculation rate
*b_Z_*	probability of human with microscopically detectable gametocytes infecting mosquito
*b_U_*	probability of human with submicroscopic gametocytes infecting mosquito
*a*	probability of human infection resulting from an infected bite
*α*	relative susceptibility to superinfection infection of Z and U
*λ*	mosquito-to-human force of infection
*Λ*	human-to-mosquito force of infection
*q*	LLIN coverage
*ε*	LLIN usage
*S*	sporozoite rate
*x*,*y*,*z*	susceptible, latent and infected mosquito subpopulations
*X*	uninfected human subpopulation
*Y*	subpopulation with asexual parasites only
*Z*	subpopulation with microscopically detectable gametocytes
*U*	subpopulation with submicroscopic gametocytes
PR_M_	microscopic asexual parasite rate
PR_S_	submicroscopic asexual parasite rate
GR_M_	microscopic gametocyte rate
GR_S_	submicroscopic gametocyte rate

**Figure 1:**
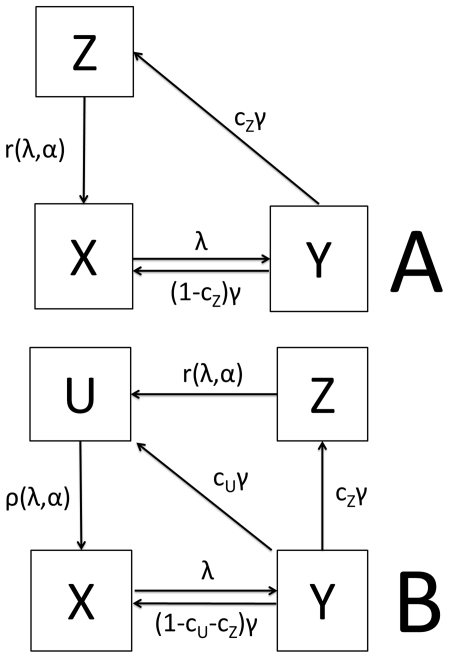
Schematic representation of the two models compared in this study. **Panel A:** Model without submicroscopic parasitemia and gametocytemia (M1). **Panel B:** Model with submicroscopic parasitemia and gametocytemia (M2). *X* – uninfected population with highest susceptibility to infection exposed to mosquito-to-human force of infection *λ*. *Y* – population with only asexual stages present, not susceptible to superinfection and developing gametocytes at a rate *γ*. A fraction of *Y* (*c_Z_*) develops microscopically detectable gametocytes. Additionally in M2, a fraction *c_U_* develops sub-microscopic gametocytes. A certain fraction of *Y* never develops detectable gametocytes (1-*c_U_*-*c_Z_*). *Z* and *U* are the populations with microscopically detectable and sub-microscopic gametocytes respectively. They have a limited susceptibility to superinfection and are exposed only to a fraction of *λ*, namely *α* (0<*α*<1). This limited susceptibility is incorporated into the models by using delayed clearance rates *r* and *ρ* for clearance of microscopically detectable and sub-microscopic gametocytes respectively. Thus, clearance rates *r* and *ρ* are functions of *λ* and *α* as well as the original clearance rates without superinfection *r_0_* and *ρ_0_* as shown in equations (10).

The model, including only data collected by LM, from now on referred to as M1, consists of three subpopulations for the human host (*X*-uninfected, *Y*- infected with only asexual parasites, *Z* – infected with asexual parasite and gametocyte forms). The model which includes observations on sub-microscopic asexual parasite and gametocyte rates, subsequently referred to as M2, has an additional infective subpopulation (*U* – infected with asexual parasites and submicroscopic gametocyte forms) representing submicroscopic gametocyte carriers with a lower probability to infect mosquitoes (*b_U_*). Both models are schematically shown in Figure 1. The differential equation systems for the human part of the models are given by equation systems (1) for M1 and (2) for M2
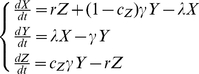
(1)

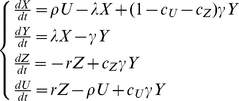
(2)In equation systems (1) and (2) the following notations are used: *λ*- mosquito-to-human force of infection, *1/γ*- latency period (duration until stage V gametocyte appearance), *r,ρ*- recovery rates of LM detectable and sub-microscopic gametocytemia, *c_Z_,c_U_* - population fraction of *Y* that move to *Z* and *U* respectively.

In both, M1 and M2, the part for the mosquito vector consists of three mosquito subpopulations (*x*-uninfected,*y*-latent,*z*-infective) with the usual transitions between them [12], [38]. The delayed differential equation system (3) describes the mosquito part of the model.
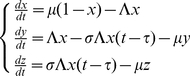
(3)In equation system (3), the following additional notations are used: *Λ* - human-to-mosquito force of infection, *μ* - mosquito mortality rate, *τ* - latency period and *σ* - mosquito survival through the extrinsic incubation period.

This mosquito part (Equations (3)) is often replaced by its quasi-equilibrated infective prevalence *S* known as the sporozoite rate and given by equation (4).
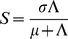
(4)The relationship between human biting rate (*h*), entomologic inoculation rate *EIR* and sporozoite rate *S* is given by equation (5).
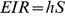
(5)


The mosquito-to-human force of infection (*λ*) and the human-to-mosquito force of infection (*Λ*) then become functions of human infectivity (*B*) and can be expressed as shown in equations (6) and (7) respectively.
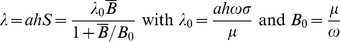
(6)

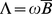
(7)In equations (6) and (7) *a* is the probability that an infectious mosquito bite results in human infection; *ω* is the mean number of bites per mosquito per day and 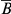 is human infectivity as defined in the next section.

### Graded human to mosquito infectivity

It has been shown that the probability of submicroscopic gametocyte carriers infecting mosquitoes is considerably reduced [22], [39]. We attempted to account for this graded human infectivity by introducing of a second infective population (*U*) into M2 representing humans with submicroscopic gametocytemia. Therefore, whereas only one infective population (*Z*) exists in M1, there are two (*Z* and *U*) in M2 representing microscopically detectable and submicroscopic gametocyte carriers, respectively. The probabilities of mosquito infection for these populations were termed *b_Z_* and *b_U_*, respectively, with *b_U_*<*b_Z_*. Thus, mean infectivity 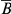 in M1 and M2 is given by equations (8) and (9), respectively.
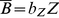
(8)

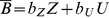
(9)


### Superinfection

To account for superinfection, the gametocyte clearance rates *r* and *ρ* in equations (1) and (2) were defined, as shown in equations (10).
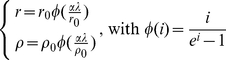
(10)In equations (10), *r_0_* and *ρ_0_* are the natural recovery rates without possibility of superinfection infection, *α* is the relative susceptibility to superinfection and *φ(i)* is the superinfection function as described by Dietz [12].

Different levels of susceptibility to superinfection were assigned to each of the human subpopulations due to gradual loss of acquired immunity after an infection. The uninfected population (*X*) is most likely to be infected and its relative susceptibility compared to the other subpopulations is equal to the value of 1. Therefore, the full *λ* is exerted on *X*. The subpopulation infected only with asexual parasites (*Y*) is assumed to be immune to superinfection and therefore has a susceptibility value of 0. The infective populations (*Z* and *U*) are susceptible to superinfection, but their recent infection provides them with greater protection. The relative susceptibility *α* for *Z* and *U* therefore lies between 0 and 1. The mosquito-to-human force of infection (*λ*) exerted on these populations then becomes *αλ*. We assume that *α* is dependent on transmission intensity. In a low transmission setting *α* is high since the population will have developed only a small degree of immunity and it is thus more likely that superinfection is successfully established after an infective bite. In a high transmission setting α is lower since the degree of immunity will be higher. A table summarizing susceptibility, infectivity and clearance for both models can be found in Appendix S1.

### Long lasting insecticide treated bed net control

For LLIN-based malaria control, the entire population was divided into a fraction ‘*q*’ benefiting from LLIN and a fraction ‘1-*q*’ not benefiting from LLIN. LLIN create a barrier between human and mosquito and therefore reduce human biting rate (*h*) for the proportion *q* of the population using LLIN. Along with *h*, mosquito force of infection (*λ*) is also is decreased, which will in turn affect clearance rates *r* and *ρ*. However the reduction in *h* is not 100%. It has been shown that use of LLIN is linked to regional, socio-economic and cultural circumstances, and to the perceived nuisance of mosquito bites and threat of malaria and other diseases [6], [40]–[42]. Therefore the parameter ‘LLIN efficiency’ (*ε*), was introduced, which determines reduction of the original *h* for the LLIN possessing part (*q*) of the population as shown in equation (11).
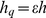
(11)The factor ‘*ε*’ can be understood as a measure of compliance with LLIN usage and incorporates the fact that not all distributed LLIN will be used to their maximum capacity. The parameters ‘*q*’ and ‘*ε*’ can be varied between 0 and 1 to mimic different malaria control scenarios. The human-to-mosquito force of infection is then represented by equation (12) where 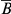 is weighted for the LLIN parameters *q* and *ε*.
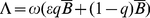
(12)Here, 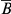 is again defined as *b_Z_Z* in M1 and by equation (9) in M2.

### Basic reproductive number

The basic reproductive number (*R_0_*) sets apart model predictions of sustained versus disrupted malaria transmission. If *R_0_*<1, a model predicts malaria eradication, otherwise sustained transmission. In the present study *R_0_* was analytically derived and computed for all model runs. LLIN usage was taken into account. Only the results by equations (13) for M1 and M2, respectively, are given (see Appendix S1 for details on the derivation of the equations).
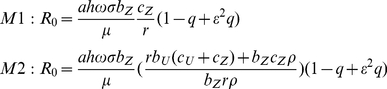
(13)The calculations in the present study were conducted using Wolfram Mathematica 7. The source codes can be downloaded by clicking on the following links http://www.cwru.edu/artsci/math/gurarie/Malaria/High%20Transmission.nb and http://www.cwru.edu/artsci/math/gurarie/Malaria/Low%20Transmission.nb for the two scenarios, respectively.

### Model calibration

The equilibrium equations for the two models along with available epidemiological data and known transmission parameters were used to estimate unknown parameters specific to a malaria-endemic environment. This section only deals with the most important aspects of model calibration. Further information and equations can be found in Appendix S1.

Some model parameters were fixed at values or within ranges published in the literature or using estimates from malaria therapy data. Table 2 shows all values which were used as fixed inputs.

**Table 2:** Fixed parameters used in the models.

Value/Unit	Value	Reference
σ [d^−1^]	0.2	[32]
μ [d^−1^]	0.16	[32], [37]
ω [d^−1^]	0.7	[35]
b_Z_	0.1–0.3	[32]
b_U_	0.01–0.1	[22], [23]
γ [d^−1^]	0.04–0.08	[59], [60]
r_0_ [d^−1^]	0.024–0.047	[53]
ρ_0_ [d^−1^]	0.024–0.047	[53]
α	0.01–0.9	arbitrary

For those values were ranges are given, we randomly sample 1000 times from the range and run model calibration for the resulting clusters of values.

Several studies have correlated probability of mosquito infection (*b_Z_* and *b_U_*) with gametocyte density. We used data from these studies to estimate the infectivity of hosts with different gametocyte density ranges. The infectivity for subpopulations *Z* and *U* was estimated from data reported by Drakeley et al., Schneider et al. and Graves et al. as *b_Z_* = 0.1–0.3 and *b_U_* = 0.01–0.1, respectively [22], [23], [32]. The gametocyte density corresponding to *b_U_* was <10 µL^−1^, which is the limit of conventional LM gametocyte detection [43].

Studies which report on microscopic and submicroscopic parasite (PR_M_ and PR_S_) and gametocyte rates (GR_M_ and GR_S_) and entomological data such as the entomological inoculation rate (EIR) are rare. We chose a low transmission setting where data were available from studies by Mwerinde (2005) and Shekalaghe et al. (2007) [24], [27] which report on all of the above parameters for an area of low transmission in Moshi, Tanzania, and a high transmission setting in Burkina Faso studied by Paganotti et al. (2004 and 2006) [25], [26].

Table 3 summarizes all values which were used from these two scenarios.

**Table 3:** Model input values from the low transmission scenario described by Shekhalage (2007) and Mwerinde (2005), and the high transmission scenario described by Paganotti (2004 and 2006) [24]–[27].

Value	Tanzania (low transmission)	Burkina Faso (high transmission)
	mean (range)*	mean (range)***
EIR (per year)	**3.4** (0.73–19.55)**	**584** (146–1022)
Parasite rate (with microscopy)	**0.019** (0.016–0.022)	**0.824** (0.742–0.9064)
Parasite rate (submicroscopic)	**0.325** (0.299–0.351)	**0.967** (0.948–0.986)
Gametocyte rate (with microscopy)	**0.004** (0.0028–0.0052)	**0.033** (0.023–0.043)
Gametocyte rate (submicroscopic)	**0.15** (0.13–0.17)	**0.41** (0.37–0.45)

Ranges were assigned to all input values. Random samples (n = 1000) were generated from within these ranges and used for model calibration.

*for parasite and gametocyte rates Shekalaghe (2007) differentiates between wet and dry season, we used the mean values;

**Mwerinde et al. report a mean EIR of 3.4/a for the Moshi region. Since this is a very low value, based on a very limited number of observations (sporozoite rate of 3/5634 sampled mosquitoes), we assume a wide (log normally distributed) possible EIR range around this mean;

***Paganotti et. al. investigated two ethnic groups (Mossi and Fulani). We used the data reported for the Fulani group.

To derive the sizes of the subpopulations (*X*,*Y*,*Z*,*U*) at endemic equilibrium from the data reported in [24]–[27], we made two assumptions: (1) all microscopically detectable parasite and gametocyte carriers are also detectable by the respective submicroscopic methods (i.e. there are no false negatives when microscopy is regarded the gold standard) and (2) all gametocyte carriers also have detectable asexual parasites and therefore contribute to the parasite rate. Furthermore M1 uses exclusively data collected by microscopy (microscopic parasite rate (PR_M_) and microscopic gametocyte rate (GR_M_)) and M2 uses data collected by microscopy and more sensitive methods (submicroscopic parasite rate (PR_S_) and submicroscopic gametocyte rate (GR_S_)). This resulted in equations (14) and (15) for M1 and M2 respectively.
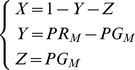
(14)

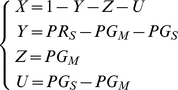
(15)Variation of the values used from [24]–[27] was allowed, based on error values reported in these studies, or based on the Poisson error, if the number of observations was reported.

The population fractions c_Z_ and c_U_ were used for calibration using the equilibrium equations of M1 and M2 (the equilibrium equations and the entire calibration scheme can be found in Appendix S1).

Little is known about the relative susceptibility (*α*). The higher the value of *α*, the more likely is superinfection in the respective population. We assume that *α* is high in a low transmission area and low in a high transmission area. Thus we chose *α* = 0.8–0.9 for model calibration with the data from [24], [27] and *α* = 0.01–0.05 for model calibration with data from [25], [26].

We did not aim to perform a best fit through more than two data sets as this was beyond the scope of the present work and very few studies report on all necessary input parameters. Furthermore the models presented here are simplistic and relatively inflexible. However, fixed and calibrated parameters should lie within reasonable boundaries.

The model calibration was run 1000 times with starting values chosen from within suitable ranges (also shown in Tables 3 and 4) to obtain the mean and a minimum to maximum range of predictions.

**Table 4:** Parameters used in M1 and M2, which were either calibrated or derived from [25]–[27].

Value	Low Transmission Scenario	High Transmission Scenario
[Unit]	mean (range)	mean (range)
*X*	**0.980** (0.978–0.984) (M1)	**0.18** (0.093–0.25) (M1)
	**0.67** (0.64–0.70) (M2)	**0.033** (0.014–0.052) (M2)
*Y*	**0.015** (0.011–0.019) (M1)	**0.79** (0.71–0.87) (M1)
	**0.17** (0.14–0.21) (M2)	**0.56** (0.50–0.61) (M2)
*Z*	**0.004** (0.002–0.005)	**0.034** (0.023–0.043)
*U*	**0.14** (0.13–0.17)	**0.38** (0.33–0.42)
*λ*	**0.00091** (0.00052–0.0014) (M1)	**0.28** (0.11–0.74) (M1)
	**0.012** (0.0092–0.019) (M2)	**0.77** (0.40–1.33) (M2)
*r* [d^−1^]	**0.030** (0.018–0.042)	**0.028** (0.016–0.043)
*ρ* [d^−1^]	**0.030** (0.018–0.042)	**0.028** (0.016–0.043)
*c_Z_*	**0.15** (0.066–0.412) (M1)	**0.024** (0.004–0.079) (M1)
	**0.014** (0.006–0.027)(M2)	**0.034** (0.012–0.078) (M2)
*c_U_*	**0.54** (0.24–0.87)	**0.36** (0.15–0.73)
*a* *	**0.12** (0.05–0.5) (M1)	**0.19** (0.05–0.5) (M1)
	**0.40**(0.05–0.5) (M2)	**0.36**(0.05–0.5) (M2)

Subpopulations *X*,*Y*,*Z* and *U* were derived from the parasite rates (PR) and gametocyte rates (GR) shown in Table 3 using equations (14) and (15). Mosquito-to-human force of infection (*λ*) was calculated using equation (S15) Clearance rates *r* and *ρ* are prolonged by superinfection as shown in equations (8). Fractions *c_Z_* and *c_U_* were used for calibration using equations (S16) and (S17) which can be found in Appendix S1.

*although a result of the calibration process (equation S16), we constrain the probability that an infective bite results in human infection (*a*) to lie between 0.05 and 0.5, which we assume to be a reasonable range for a highly uncertain parameter such as *a*.

Parasite Rate (PR) in our plots corresponds to either *Y+Z* in M1 or *Y+Z+U* in M2. The cut-off PR that was set to indicate malaria elimination was 10^−4^ (<1 case of malaria in 10,000 people).

## Results

The basic reproductive number (*R_0_*), which is essential for sustained infection, showed marked differences between M1 and M2 in both high and low transmission settings. The predicted *R_0_* at endemic equilibrium (without LLIN control) was also considerably higher for M2 in both the low (*R_0_* for M1: 1.001–1.025 vs. *R_0_* for M2: 1.47–1.64) and high (*R_0_* for M1: 3.8–10.4 vs. *R_0_* for M2: 20.6–57.00) transmission settings. Because of the different stability levels of the endemic equilibria in the two models, similar malaria control efforts will have different, model-dependent, predicted impacts. While a defined control attempt could disrupt transmission for M1, bringing *R_0_* to values <1 (unsustainable transmission), M2 could still predict persistent transmission at another equilibrium state with *R_0_*>1.

**Figure 2:**
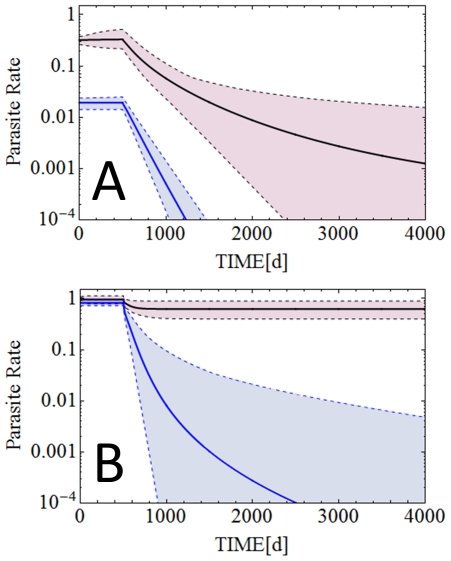
Model predictions for different LLIN based control interventions. **Panel A:** Equilibrium parasite rates and their changes with a LLIN intervention starting at t = 500 days, with 50% LLIN coverage and 40% LLIN usage as predicted by M1 and M2 respectively for the low transmission setting described by Shekalaghe et al. and Mwerinde et al. [24], [27]. **Panel B:** Predictions of M1 and M2 when LLIN coverage is 92% and LLIN usage is 88% in the high transmission scenario as described by Paganotti et al. [25], [26].

Figure 2 shows specific examples for cases of malaria control with LLIN in which M1 predicts malaria eradication while M2 predicts persistent transmission. Figure 2A shows the scenario of low transmission with LLIN coverage of 50% and usage of 40%. These values resemble LLIN coverage and usage achieved across Africa and other endemic areas in recent years [5], [6]. In this case M1 predicts disrupted malaria transmission and malaria eradication in 1.9 years (*R_0_* is reduced to 0.68–0.69 in M1), whereas M2 predicts persistent transmission at a lower level (*R_0_* is reduced to 1.00–1.11 in M2). In the high transmission setting these values of LLIN coverage and usage do not result in predicted malaria eradication by either M1 or M2. Therefore, a hypothetical control scenario set in the high transmission environment is presented in Figure 2B, in which LLIN coverage is 92% and LLIN usage is 88%. In this case M1 predicts malaria eradication from the high transmission area in approx. 6.8 years (*R_0_* 0.35 to 0.97 is reduced to in M1), whereas M2 only predicts a sustained 30% reduction in malaria prevalence (*R_0_* is reduced to 1.9–5.3 in M2).

**Figure 3:**
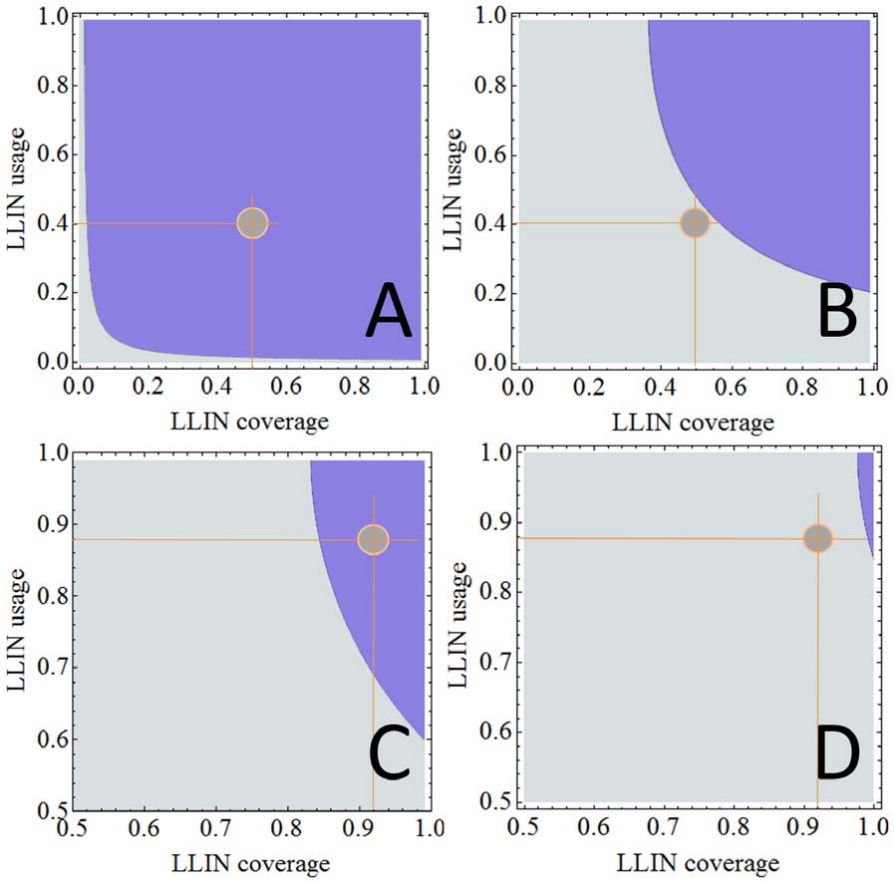
Basic reproduction numbers (*R_0_*). Basic reproductive numbers (*R_0_*) for all values of LLIN coverage and usage for M1 (Panel A) and M2 (Panel B) in the low transmission setting and M1 (Panel C) and M2 (Panel D) in the high transmission setting. Light blue areas denote *R_0_*>1 and dark blue areas denote *R_0_*<1. The light brown circles denote the scenarios explicitly shown in Figure 2. Note that *R_0_* = 1 sets apart ‘sustained transmission’ from ‘malaria eradication’. It can clearly be seen that in Panels A and C (M1) *R_0_* is well below this discriminating value of 1. In Panels B and D (M2) the values for *R_0_* are clearly below the value of 1. Therefore, while M1 predicts malaria eradication, M2 still predict persistent transmission.

Whereas Figure 2 shows parasite rates for specific malaria control scenarios, Figure 3 shows *R_0_* for all possible combinations of LLIN coverage (0<q<1) and LLIN usage (0<ε<1) calculated using equations (13). It can be seen that much higher levels of LLIN coverage and usage are required as predicted by M2 compared to M1 to reduce *R_0_* to values below 1.

## Discussion

The present study emphasizes the important role that host reservoirs with low-level gametocyte and asexual parasite density can play in sustaining malaria transmission despite LLIN-based malaria control. For any given control scenario, the model which includes a large pool of hosts with sub-microscopic gametocyte and asexual parasite densities predicts considerably higher transmission and therefore a need for greater coverage and adherence to LLIN usage. When LLIN coverage is set to levels at or around values currently achieved with the up-scaled efforts enabled by The Global Fund to Fight AIDS, Tuberculosis and Malaria (GFATM) [5], only M1 in a low transmission setting predicts malaria eradication. M2 does not predict interrupted transmission even in the low transmission setting (Figure 2A). For high transmission environments, the models presented here predict the need for much higher LLIN coverage and usage. Furthermore our predictions emphasize the importance of implementing sensitive diagnostic approaches that are used for monitoring and evaluating malaria control strategies and the progress of malaria control campaigns.

Although previous models acknowledge submicroscopic gametocytemia [11], [44], our current approach is, to the best of our knowledge, the first to evaluate the impact of these infections on malaria transmission and control. In our models, LLIN is the only malaria control strategy. In real-life field situations, efficient malaria control utilizes LLIN as part of a range of co-ordinated strategies. The next phase of worldwide intensified malaria control aims to build on the success of LLIN distribution in 2008 by deploying rapid diagnostic tests and effective antimalarial therapies as widely as possible in order to reduce preventable deaths from malaria to near zero by 2015 [5]. It should be noted that efficient treatment of symptomatic malaria cases, while undoubtedly an urgent priority, will only partially reduce malaria transmission because asymptomatic carriers will continue to transmit the disease'.

Adult populations in holo- or hyperendemic regions have high levels of acquired immunity to the severe manifestations of the infection. Although parasitemic, these people do not usually feel ill and thus see no reason to visit a health center or seek treatment, but they are likely to harbor infectious gametocytes that will sustain malaria transmission regardless of a successful local reduction in symptomatic malaria. Asymptomatic malaria infection is normally characterized by very low asexual parasite densities and the absence of gametocytes, especially in adults [45]–[47]. A recent review by Okell et al. [17] revealed that standard LM failed to detect asexual malaria parasites in 49.2% of all cases and 91.3% of gametocytemic cases. Furthermore, Bonnet et al. estimate the contribution of hosts with undetectable gametocytemia to malaria transmission at 23.7% [21], [22] while Schneider et al. determined an even higher value at 54.8% [21], [22]. Sustained active case detection using rapid diagnostic tests and LM will, therefore, be inadequate because the sensitivity of these methods is too low. It is important to maximize detection of cases infectious to mosquitoes for a prolonged period, presumably decades, to achieve true malaria eradication [48].

If a fraction of malaria cases go undetected and the intensified efforts of malaria control are reduced prematurely, malaria will increase such as it did in India [49], Sri Lanka [50], Zanzibar [50] and Madagascar [51] after the halt of the eradication programs in the last century. Resurgent malaria can have devastating effects on populations with levels of immunity that have waned following partially effective control. For example, the incidence of malaria in the highlands of Madagascar fell dramatically during the control program in the 20^th^ century but more than 40,000 fatalities were attributed to malaria during the 5 years after the control program was stopped [51].

It will be very important to complement current methods of surveillance with enhanced diagnostic techniques to more accurately plan, monitor progress and guide malaria control which is aimed at eliminating malaria. Recently developed methods to detect gametocytes at low densities such as RT-PCR, QT-NASBA and HFGMF can reduce the threshold of gametocyte detection to cover a wider epidemiologically relevant range. Application of these methods and their potential refinement to maximize time and cost effectiveness appears a vital component of successful malaria elimination.

It is particularly important to detect gametocytes because they are more resilient to treatment. While there is evidence of a gametocytocidal effect of artemisinin drugs, gametocyte clearance times are still very long in ACT-treated patients with a mean of 49 h for asexual stages and 220 h (>9 days) for gametocytes in a study of 559 patients conducted in Thailand in from 1998 to 2006 [52]. Another recent study detected submicroscopic gametocytes up to 48 days after ACT treatment [53]. Primaquine, the most potent gametocytocidal drug, can provoke hemolysis in glucose-6-phosphate dehydrogenase (G-6-PD) deficient patients and is thus not used routinely in the many tropical countries in which G-6-PD deficiency has been observed [54], [55]. This means that some patients can be infective for weeks following clearance of asexual parasites even after ACT treatment. ACT or other treatments were not included in the current analyses, in part because of factors such as drug-specific post-treatment gametocyte viability [56], [57] and a variable if brief period of carriage of gametocytes without asexual forms. We believe that the problem of submicroscopic gametocytemia sustaining malaria transmission is mostly relevant in asymptomatic malaria carriers. Since they will rarely seek treatment, ACT/other therapy will not have a great impact on this part of the population. The exception may be mass drug administration which is not likely to play a big role in malaria control efforts, especially with the fear of emerging artemisinin resistance [58].

The aim of the present study was to highlight the importance of sensitive gametocyte and asexual parasite detection. For this we have used data from two very different transmission settings to calibrate the models. Other calibration procedures that constrain the range of admissible parameters could also be used. The models presented here have several limitations. Ross-Macdonald methodology is a very simplistic approach, since the real-world dynamics of malaria infection depend on many more variables (such as the age distribution of the population, seasonality of transmission and protective host factors including red cell polymorphisms) that cannot be encompassed by such models. Nevertheless, our adaptation of the most commonly used form of mathematical models for malaria can be viewed as a necessary refinement in their further development and an important step in identifying practical strategies for parasite detection necessary to enhance control and elimination efforts.

## Supporting Information

Appendix S1 **The appendix contains more detailed information on equilibrium equations, model calibration, graded susceptibility and infectivity and the analytical derivation of the basic reproductive numbers.**
(DOC)

## References

[1] Bhattarai A, Ali AS, Kachur SP, Martensson A, Abbas AK (2007). Impact of artemisinin-based combination therapy and insecticide-treated nets on malaria burden in Zanzibar.. PLoS Med.

[2] Nyarango PM, Gebremeskel T, Mebrahtu G, Mufunda J, Abdulmumini U (2006). A steep decline of malaria morbidity and mortality trends in Eritrea between 2000 and 2004: the effect of combination of control methods.. Malar J.

[3] Otten M, Aregawi M, Were W, Karema C, Medin A (2009). Initial evidence of reduction of malaria cases and deaths in Rwanda and Ethiopia due to rapid scale-up of malaria prevention and treatment.. Malar J.

[4] Hommel M (2008). Towards a research agenda for global malaria elimination.. Malar J.

[5] Chambers RG (2009). United Nations Secretary-General's Special Envoy for Malaria Progress Report.

[6] Atkinson JA, Bobogare A, Fitzgerald L, Boaz L, Appleyard B (2009). A qualitative study on the acceptability and preference of three types of long-lasting insecticide-treated bed nets in Solomon Islands: implications for malaria elimination.. Malar J.

[7] Ross R (1911). The prevention of Malaria.

[8] Macdonald G (1952). The analysis of equilibrium in malaria.. Trop Dis Bull.

[9] Smith DL, Hay SI (2009). Endemicity response timelines for *Plasmodium falciparum* elimination.. Malar J.

[10] Anderson RM, May RM, Anderson B (1991). Infectious diseases of humans.

[11] Aron JL, May RM, Anderson RM (1982). The population dynamics of malaria.. Population Dynamics of Infectious Diseases: Theory and Applications.

[12] Bailey NJT (1982). The Biomathematics of Malaria.

[13] Dietz K, Molineaux L, Thomas A (1974). A malaria model tested in the African savannah.. Bull World Health Organ.

[14] Koella JC (1991). On the use of mathematical models of malaria transmission.. Acta Trop.

[15] Koella JC, Antia R (2003). Epidemiological models for the spread of anti-malarial resistance.. Malar J.

[16] Molineaux L, Gramiccia G (1980). The Garki Project.

[17] Okell LC, Ghani AC, Lyons E, Drakeley CJ (2009). Submicroscopic infection in *Plasmodium falciparum*-endemic populations: a systematic review and meta-analysis.. J Infect Dis.

[18] Schneider P, Bousema T, Omar S, Gouagna L, Sawa P (2006). (Sub)microscopic *Plasmodium falciparum* gametocytaemia in Kenyan children after treatment with sulphadoxine-pyrimethamine monotherapy or in combination with artesunate.. Int J Parasitol.

[19] Karl S, David M, Moore L, Grimberg BT, Michon P (2008). Enhanced detection of gametocytes by magnetic deposition microscopy predicts higher potential for *Plasmodium falciparum* transmission.. Malar J.

[20] Karl S, Davis TME, St. Pierre TG (2009). A comparison of the sensitivities of detection of *Plasmodium falciparum* gametocytes by magnetic fractionation, thick blood film microscopy, and RT-PCR.. Malar J.

[21] Bonnet S, Gouagna LC, Paul RE, Safeukui I, Meunier JY (2003). Estimation of malaria transmission from humans to mosquitoes in two neighbouring villages in south Cameroon: evaluation and comparison of several indices.. Trans R Soc Trop Med Hyg.

[22] Schneider P, Bousema JT, Gouagna LC, Otieno S, van de Vegte-Bolmer M (2007). Submicroscopic *Plasmodium falciparum* gametocyte densities frequently result in mosquito infection.. Am J Trop Med Hyg.

[23] Drakeley CJ, Secka I, Correa S, Greenwood BM, Targett GA (1999). Host haematological factors influencing the transmission of *Plasmodium falciparum* gametocytes to Anopheles gambiae s.s. mosquitoes.. Trop Med Int Health.

[24] Mwerinde O, Oesterholt M, Harris C, Lushino P, Masokoto A (2005). Monitoring seasonal variations in malaria transmission in a lowendemicity area in Tanzania, MIM Abstract 101B.. Acta Tropica.

[25] Paganotti G, Babiker H, Modiano D, Sirima B, Verra F (2004). Genetic complexity of *Plasmodium falciparum* in two ethnic groups of Burkina Faso with marked differences in susceptibility to malaria.. American Journal of Tropical Medicine and Hygiene.

[26] Paganotti G, Palladino C, Modiano D, Sirima B, Radberg L (2006). Genetic complexity and gametocyte production of *Plasmodium falciparum* in Fulani and Mossi communities in Burkina Faso.. Parasitology.

[27] Shekalaghe S, Bousema T, Kunei K, Lushino P, Masokoto A (2007). Submicroscopic *Plasmodium falciparum* gametocyte carriage is common in an area of low and seasonal transmission in Tanzania.. Tropical Medicine and International Health.

[28] Collins WE, Jeffery GM (1999). A retrospective examination of the patterns of recrudescence in patients infected with *Plasmodium falciparum*.. Am J Trop Med Hyg.

[29] Collins WE, Jeffery GM (1999). A retrospective examination of sporozoite- and trophozoite-induced infections with *Plasmodium falciparum* in patients previously infected with heterologous species of Plasmodium: effect on development of parasitologic and clinical immunity.. Am J Trop Med Hyg.

[30] Collins WE, Jeffery GM (1999). A retrospective examination of secondary sporozoite- and trophozoite-induced infections with *Plasmodium falciparum*: development of parasitologic and clinical immunity following secondary infection.. Am J Trop Med Hyg.

[31] Collins WE, Jeffery GM (1999). A retrospective examination of sporozoite- and trophozoite-induced infections with *Plasmodium falciparum*: development of parasitologic and clinical immunity during primary infection.. Am J Trop Med Hyg.

[32] Graves PM, Burkot TR, Carter R, Cattani JA, Lagog M (1988). Measurement of malarial infectivity of human populations to mosquitoes in the Madang area, Papua, New Guinea.. Parasitology.

[33] Burkot TR, Dye C, Graves PM (1989). An analysis of some factors determining the sporozoite rates, human blood indexes, and biting rates of members of the Anopheles punctulatus complex in Papua New Guinea.. Am J Trop Med Hyg.

[34] Burkot TR, Graves PM, Paru R, Wirtz RA, Heywood PF (1988). Human malaria transmission studies in the Anopheles punctulatus complex in Papua New Guinea: sporozoite rates, inoculation rates, and sporozoite densities.. Am J Trop Med Hyg.

[35] Canyon DV, Hii JL, Muller R (1999). Effect of diet on biting, oviposition, and survival of Aedes aegypti (Diptera: Culicidae).. J Med Entomol.

[36] Cattani JA, Moir JS, Gibson FD, Ginny M, Paino J (1986). Small-area variations in the epidemiology of malaria in Madang Province.. P N G Med J.

[37] Le Menach A, McKenzie FE, Flahault A, Smith DL (2005). The unexpected importance of mosquito oviposition behaviour for malaria: non-productive larval habitats can be sources for malaria transmission.. Malar J.

[38] Anderson RM, May RM, Gupta S (1989). Non-linear phenomena in host-parasite interactions.. Parasitology.

[39] Jefferey G, Eyles D (1955). Infectivity to mosquitoes of plasmodium falicparum as related to gametocyte density and duration of infection.. Am J Trop Med Hyg.

[40] MacCormack CP, Snow RW (1986). Gambian cultural preferences in the use of insecticide-impregnated bed nets.. J Trop Med Hyg.

[41] Okrah J, Traore C, Pale A, Sommerfeld J, Muller O (2002). Community factors associated with malaria prevention by mosquito nets: an exploratory study in rural Burkina Faso.. Trop Med Int Health.

[42] Toe LP, Skovmand O, Dabire KR, Diabate A, Diallo Y (2009). Decreased motivation in the use of insecticide-treated nets in a malaria endemic area in Burkina Faso.. Malar J.

[43] Bejon P, Andrews L, Hunt-Cooke A, Sanderson F, Gilbert S (2006). Thick blood film examination for *Plasmodium falciparum* malaria has reduced sensitivity and underestimates parasite density.. Malar J.

[44] Filipe JAN, Riley EM, Drakeley CJ, Sutherland CJ, Ghani AC (2007). Determination of the Processes Driving, the Acquisition of Immunity to Malaria Using, a Mathematical Transmission Model.. PloS Computational Biology.

[45] Owusu-Agyei S, Binka F, Koram K, Anto F, Adjuik M (2002). Does radical cure of asymptomatic *Plasmodium falciparum* place adults in endemic areas at increased risk of recurrent symptomatic malaria?. Trop Med Int Health.

[46] Owusu-Agyei S, Koram KA, Baird JK, Utz GC, Binka FN (2001). Incidence of symptomatic and asymptomatic *Plasmodium falciparum* infection following curative therapy in adult residents of northern Ghana.. Am J Trop Med Hyg.

[47] Bousema JT, Gouagna LC, Drakeley CJ, Meutstege AM, Okech BA (2004). *Plasmodium falciparum* gametocyte carriage in asymptomatic children in western Kenya.. Malar J.

[48] Feachem R, Phillips A, Targett G (2009).

[49] Sharma VP, Mehrotra KN (1986). Malaria resurgence in India: a critical study.. Soc Sci Med.

[50] Curtis CF (2002). Should the use of DDT be revived for malaria vector control?. Biomedica.

[51] Romi R, Razaiarimanga MC, Raharimanga R, Rakotondraibe EM, Ranaivo LH (2002). Impact of the malaria control campaign (1993–1998) in the highlands of Madagascar: parasitological and entomological data.. Am J Trop Med Hyg.

[52] Tangpukdee N, Krudsood S, Srivilairit S, Phophak N, Chonsawat P (2008). Gametocyte clearance in uncomplicated and severe *Plasmodium falciparum* malaria after artesunate-mefloquine treatment in Thailand.. Korean J Parasitol.

[53] Bousema T, Okell L, Shekalaghe S, Griffin JT, Omar S (2010). Revisiting the circulation time of *Plasmodium falciparum* gametocytes: molecular detection methods to estimate the duration of gametocyte carriage and the effect of gametocytocidal drugs.. Malar J.

[54] Tarlov AR, Brewer GJ, Carson PE, Alving AS (1962). Primaquine sensitivity. Glucose-6-phosphate dehydrogenase deficiency: an inborn error of metabolism of medical and biological significance.. Arch Intern Med.

[55] Baird JK, Hoffman SL (2004). Primaquine therapy for malaria.. Clin Infect Dis.

[56] Beavogui AH, Djimde AA, Gregson A, Toure AM, Dao A (2010). Low infectivity of *Plasmodium falciparum* gametocytes to Anopheles gambiae following treatment with sulfadoxine-pyrimethamine in Mali.. Int J Parasitol.

[57] Kone A, van de Vegte-Bolmer M, Siebelink-Stoter R, van Gemert GJ, Dara A (2010). Sulfadoxine-pyrimethamine impairs *Plasmodium falciparum* gametocyte infectivity and Anopheles mosquito survival.. Int J Parasitol.

[58] Dondorp AM, Nosten F, Yi P, Das D, Phyo AP (2009). Artemisinin resistance in *Plasmodium falciparum* malaria.. N Engl J Med.

[59] Murphy JR, Baqar S, Davis JR, Herrington DA, Clyde DF (1989). Evidence for a 6.5-day minimum exoerythrocytic cycle for *Plasmodium falciparum* in humans and confirmation that immunization with a synthetic peptide representative of a region of the circumsporozoite protein retards infection.. J Clin Microbiol.

[60] Talman AM, Domarle O, McKenzie FE, Ariey F, Robert V (2004). Gametocytogenesis: the puberty of *Plasmodium falciparum*.. Malar J.

