# Supplementary material for: A Sub-Microscopic Gametocyte Reservoir Can Sustain Malaria Transmission
Source: PLoS One. 2011 Jun 14;6(6):e20805. doi: 10.1371/journal.pone.0020805 (PMC3114851; doi:10.1371/journal.pone.0020805)
Supplement: Appendix S1 — The appendix contains more detailed information on equilibrium equations, model calibration, graded susceptibility and infectivity and the analytical derivation of the basic reproductive numbers. (DOC) [file pone.0020805.s001.doc]

# Appendix S1

## Equilibrium Equations

For M1 the explicit solution for the equilibrium state (*X’(t)*=*Y’(t)*=*Z’(t)*=0) is given by equation (S1) to (S3).

$X=\frac{r\gamma}{c_{Z}\gamma\lambda+r(\lambda+\gamma)}$ (S1)

$Y=\frac{r\lambda}{c_{Z}\gamma\lambda+r(\lambda+\gamma)}$ (S2)

$Z=\frac{c_{Z}\gamma\lambda}{c_{Z}\gamma\lambda+r(\lambda+\gamma)}$ (S3)

Similarly, for M2 the explicit solution for the equilibrium state (*X’(t)*=*Y’(t)*=*Z’(t)*=*U’(t)*=0) is given by equation (S4) to (S8).

$X=\frac{r\gamma\rho}{c_{U}r\gamma\lambda+r\rho(\gamma+\lambda)+c_{Z}\gamma\lambda(r+\rho)}$ (S4)

$Y=\frac{r\lambda\rho}{c_{U}r\gamma\lambda+r\rho(\gamma+\lambda)+c_{Z}\gamma\lambda(r+\rho)}$ (S5)

$Z=\frac{c_{Z}\gamma\lambda\rho}{c_{U}r\gamma\lambda+r\rho(\gamma+\lambda)+c_{Z}\gamma\lambda(r+\rho)}$ (S6)

$U=\frac{(c_{U}+c_{Z})r\gamma\lambda}{c_{U}r\gamma\lambda+r\rho(\gamma+\lambda)+c_{Z}\gamma\lambda(r+\rho)}$ (S7)

## Model calibration

Calibration was conducted for a low and a high transmission scenario. Data to calculate the size of subpopulations *X*, *Y*, *Z* and *U* was taken from [1-4] and is shown in Table 3 of the main text. It was assumed that all microscopically detectable parasite and gametocyte carriers are also detectable by submicroscopic methods and that all gametocyte carriers also have detectable asexual parasites. Furthermore M1 uses exclusively data collected by microscopy (microscopic parasite rate (PR_M_) and microscopic gametocyte rate (GR_M_)) and M2 uses data collected by microscopy and more sensitive methods (submicroscopic parasite rate (PR_S_) and submicroscopic gametocyte rate (GR_S_)). Therefore the following relations were used to calculate the sizes of subpopulations *X*,*Y*,*Z* and *U* at endemic equilibrium. For M1 as in equations (S8)-(S10).

*X=1-Y-Z* (S8)

*Y=PR_M_-PG_M_* (S9)

*Z=PG_M_* (S10)

For M2 as in equations (S11)-(S14).

*X=1-Y-Z-U* (S11)

*Y=PR_S_-PG_M_-PG_S_* (S12

*Z=PG_M_* (S13)

*U=PG_S_-PG_M_* (S14)

The mosquito-to-human force of infection (*λ*) was calculated from the equilibrium state as shown in equation S15.

$\lambda=\frac{\gamma Y}{X}$ (S15)

The fractions *c_Z_* and *c_U_* were also used for calibration and calculated using the following relations.

$c_{Z}=\frac{rZ}{\gamma Y}$ (S16)

$c_{U}=\frac{\rho U-rZ}{\gamma Y}$ (S17)

The probability ‘*a*’, that an infected bite truly results in mosquito infection is calculated by equation S16.

$a=\frac{\lambda}{EIR}$ (S16)

## Basic reproductive number (R_0_)

The infectivity *B* of each subpopulation is given by the probability to infect a mosquito multiplied by the size of the subpopulation. In M1 only subpopulation *Z* can infect mosquitoes with the probability *b_Z_*. Therefore the infectivity B in M1 is given by equation (S17).

$B=b_{Z}Z$ (S17)

The equilibrium equation for *Z* (S7) is now substituted for *Z* in equation (S17) to yield equation (S18).

$B=f[B]=\frac{b_{Z}c_{Z}\gamma\lambda[B]}{c_{Z}\gamma\lambda[B]+r[B](\lambda[B]+\gamma)}$, (S18)

The parameters *λ* and *r* are also functions of *B* as shown in equations (S19) and (S20)

$\lambda[B]=\frac{ah\omega\sigma B}{\mu(1+\frac{\mu B}{\omega})}$ (S19)

and

$r[B]=r_{0}\varphi(\frac{\alpha\lambda[B]}{r_{0}})$ with $\varphi[i]=\frac{1}{e^{i}-1}$ (S20)

The solvability condition for *f[B]* >0 is *f’(0)*=*R_0_*>1, which can be found by conversion of (S17) into a polynomial series and differentiation. This was done using Wolfram Mathematica 7.0 and the result is shown in equation (S21).

$R_{0}=f'(0)=\frac{c_{Z}b_{Z}ah\omega\sigma}{\mu r}>1$ (S21)

*R_0_* can be calculated similarly for M2. There are now two infective subpopulations *Z* and *U*. These are weighted with their respective infection probabilities. Thus, in M2, *B* becomes equation (S21).

$B=b_{Z}Z+b_{U}U$ (S22)

In equation (S21), *Z* and *U* are again substituted by the equilibrium equations (S6) and (S7) and *λ*, *r* and *ρ* are functions of *B* as shown in equations (S19), (S20) and (S23).

$\rho[B]=\rho_{0}\varphi(\frac{\alpha\lambda[B]}{\rho_{0}})$ with $\varphi[i]=\frac{1}{e^{i}-1}$ (S23)

The solution for *f’(0)*=*R_0_*>1 can be found by conversion of (S22) into a polynomial series and differentiation and is shown in equation (S24).

$R_{0}=\frac{ah\omega\sigma}{\mu}(\text{ }\frac{rb_{U}(c_{U}+c_{Z})+b_{Z}c_{Z}\rho}{b_{Z}r\rho})$ (S24)

## R_0_ for LLIN coverage

The entire population was split into a LLIN covered fraction(*q*) and an non covered fraction (1-*q*). The mosquito force of infection (*λ)* for the non-covered fraction remains the same as in the models without LLIN. For the covered population *λ* is reduced to *λ_q_=ελ* where *ε* is the degree LLIN usage. The LLIN covered subpopulations are now designated by the index ‘*q*’, *X_q_,Y_q_,Z_q_* and *U_q_*. The human-to-mosquito force of infection (*Λ*) is then given by equation (S25).

$\Lambda=\omega(q\varepsilon B_{C}+(1-q)B)$, (S25)

In M1, infectivity for the non-covered fraction of the population again corresponds to $B=b_{Z}Z$ and for the covered population infectivity corresponds to$B_{C}=b_{Z}Z_{C}$.

Similarly to before *R_0_* can now be calculated by the substitution of *Z* and *Z_C_* by their equilibrium equations as shown in equations S26 and S27.

$B=f[B]=\frac{c_{Z}\gamma\lambda[B]}{c_{Z}\gamma\lambda[B]+r[B](\lambda[B]+\gamma)}$ (S26)

$B_{C}=f[B_{C}]=\frac{c_{Z}\gamma\lambda[B_{C}]}{c_{Z}\gamma\lambda[B_{C}]+r[B_{C}](\lambda[B_{C}]+\gamma)}$ (S27)

The functions $f\left[B\right]$and $f\left[B_{C}\right]$ are now inserted into equation (S25) for *B* and *B_C_*.

The solvability condition for Λ is $\Lambda'\left(0\right)=R_{0}>1$.The solution is given by equation (S28) and represents a scaling factor of the original *R_0_* (without LLIN).

$R_{0}^{LLIN}=(1-q+\varepsilon^{2}q)R_{0}$ (S28)

For M2 the derivation is analogous and equation S28 applies to M1 and M2. Please note that *R_0_* in (S28) is the original *R_0_* without LLIN (Equation (S21) for M1 and (S24) in M2)). Therefore we skip the details for M2 here.

## Susceptibility, infectivity, human force of infection and clearance for human sub-populations

Table S1: Summary of susceptibility, infectivity, mosquito-tohuman force of infection and clearance of the different populations (X,Y,Z,U)

| subpopulation | susceptibility | infectivity | mosquito-to-human force of infection | clearance |
| --- | --- | --- | --- | --- |
| X | 1 | 0 | λ  | - |
| Y | 0 | 0 | −  | γ  |
| Z | α  | b_Z_ | α λ | r |
| U | α  | b_U_ | α λ | ρ |

## Model Codes:

The model codes which were developed and used in the present study can be accessed by clicking below links. We used Wolfram Mathematica version 7.0.

[http://www.cwru.edu/artsci/math/gurarie/malaria/](http://www.cwru.edu/artsci/math/gurarie/Diabetes/diabetes Model outline.htm)M1.nb and [http://www.cwru.edu/artsci/math/gurarie/malaria/](http://www.cwru.edu/artsci/math/gurarie/Diabetes/diabetes Model outline.htm)M2.nb.

## References

1. O. Mwerinde, M. Oesterholt, C. Harris et al., Acta Tropica 95S, 183 (2005).

2. G. Paganotti, H. Babiker, D. Modiano et al., American Journal of Tropical Medicine and Hygiene 71 (2) (2004).

3. G. Paganotti, C. Palladino, D. Modiano et al., Parasitology 2006 (132) (2006).

4. S. Shekalaghe, T. Bousema, K Kunei et al., Tropical Medicine and International Health 12 (2007).
